# Combined Fundal and Segmental Adenomyomatosis of the Gallbladder in a Child: A Rare Case Report

**DOI:** 10.1155/2019/2659089

**Published:** 2019-11-28

**Authors:** Hidetoshi Kinoshita, Hiromichi Ariga, Jun Shirota, Kyosuke Sasaki, Yasuko Shibukawa, Yutaka Fukuda, Katsutoshi Nagasawa, Seiya Ogata, Hirofumi Shimizu, Michitoshi Yamashita, Hideaki Tanaka

**Affiliations:** ^1^Department of Pediatrics, Takeda General Hospital, 3-27 Yamaga-machi, Aizuwakamatsu City, Fukushima 965-8585, Japan; ^2^Department of Pediatric Surgery, Fukushima Medical University Hospital, 1 Hikarigaoka, Fukushima City, Fukushima 960-1247, Japan

## Abstract

Adenomyomatosis of the gallbladder (AMG) is characterized by mucosal hyperplasia leading to invagination through the thickened muscle layer, which is relatively common in adults, but is rare in childhood. We report a 12-year-old boy with adenomyomatosis of the gallbladder combined segmental and fundal type. This combined type is rare in adults and is first reported here in childhood. Although initial imaging with computed tomography (CT) suggested the presence of a circular solid mass-like lesion because of its rare morphology, repeated ultrasonography (US) was useful for leading to a correct diagnosis.

## 1. Introduction

Adenomyomatosis of the gallbladder (AMG) is a benign and acquired lesion characterized by mucosal hyperplasia leading to invagination through the thickened muscle layer, known as the Rokitansky–Aschoff sinuses (RAS) [1]. While AMG is relatively common in adults, which incidentally discovered in up to 5% of cholecystectomy specimens, few pediatric cases have been reported in the literature. AMG has generally been classified into three types: diffuse, segmental, and fundal. We herein present a very rare case with AMG of combined segmental and fundal type, in which initial imaging with computed tomography (CT) suggested the presence of a circular solid mass-like lesion because of its rare morphology.

## 2. Case Presentation

A 12-year-old boy with acute abdominal pain presented to our hospital. The patient reported that pain sometimes occurred postprandially, and that the frequency and severity had worsened over the previous six months.

**Figure 1:**
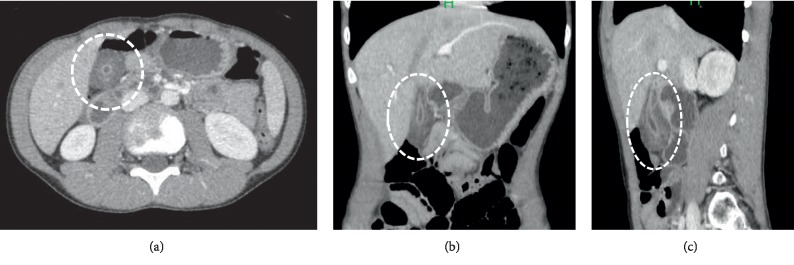
Abdominal contrast-enhanced computed tomography revealed a circular solid mass-like lesion (circle) near the normal-looking gallbladder on admission: (a) axial view, (b) coronal view, and (c) sagittal view.

**Figure 2:**
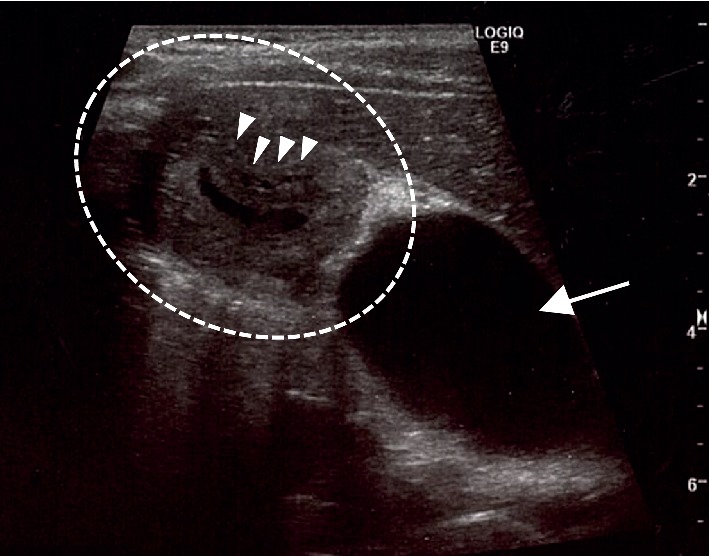
Repeated ultrasonography the next day of admission revealed that the proximal dilated gallbladder (arrow) was continuous with the mass (circle), the wall of which contained several small cysts (arrowheads).

**Figure 3:**
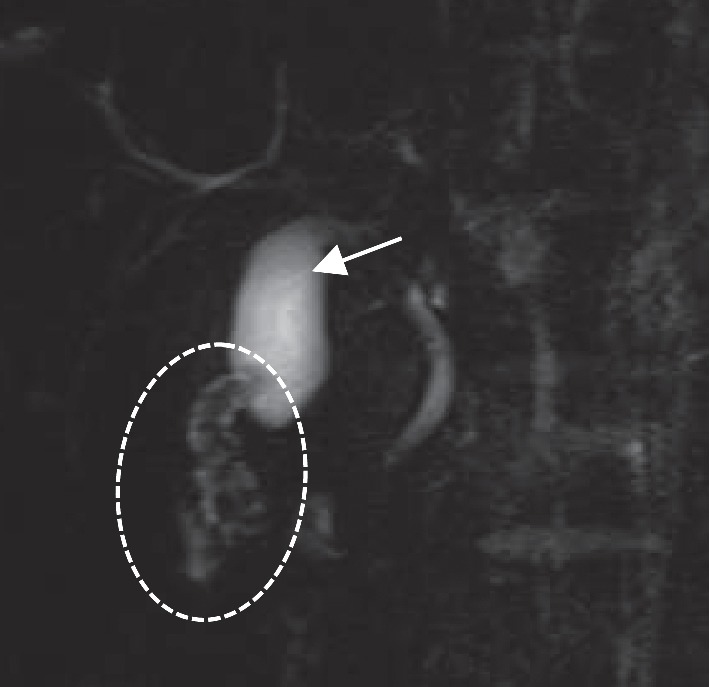
Magnetic resonance cholangiopancreatography revealed a pearl necklace sign (circle), continuous with the proximal gallbladder (arrow), and no other anomalies (e.g., pancreaticobiliary maljunction).

**Figure 4:**
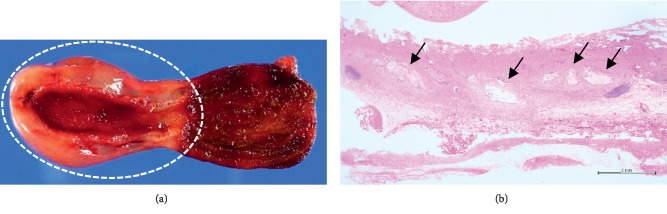
(a) More than half of the distal wall of the gallbladder was thickened (circle). (b) A histopathological examination revealed that the thickened wall contained Rokitansky–Aschoff sinuses (arrows) combined with hyperplasia of the smooth muscle and collagen fibers, which were compatible with adenomyomatosis of the gallbladder.

A physical examination revealed normal findings, with the exception of the right upper abdominal tenderness. The laboratory data were within normal limits (data not shown). Contrast-enhanced CT in axial view in the first radiologic examination showed a circular solid mass-like lesion (30 × 23 mm in diameter) adjacent to the liver that was heterogeneously enhanced with a small luminal structure in the center (Figure 1(a)). The association between the mass and the gallbladder was not clearly visualized. A careful US examination led us to suspect that the mass was part of the gallbladder. The patient's abdominal pain improved on the day after admission and fasting. The second US examination revealed that the mass-like lesion was actually the thickened wall of the body and fundus of the gallbladder, which contained several small cysts and a small lumen in its center. It was continuous to the expanded normal gallbladder wall (Figure 2). The first CT was reconstructed in coronal and sagittal views, and the lesion was recognized as a thickened wall of the entire gallbladder (Figures 1(b) and 1(c)). Magnetic resonance cholangiopancreatography (MRCP) revealed small cysts with an orderly alignment and high-intensity signals in the thickened wall of the body and fundus of the gallbladder, which was regarded as the pearl necklace sign of AMG (Figure 3), and showed no other abnormalities (e.g., pancreaticobiliary maljunction [PBM]). He was finally diagnosed with AMG and underwent laparoscopic cholecystectomy. The macroscopic observation of the resected gallbladder revealed thickening of more than distal half of the body. The wall of the middle of the body was far more thickened than that of the peripheral area, causing slight stenosis (Figure 4(a)). The histopathological findings of all areas of the thickened wall contained Rokitansky–Aschoff sinuses (RASs) combined with hyperplasia of the smooth muscle and collagen fibers (Figure 4(b)), which were compatible with AMG, and no malignant or premalignant findings. Based on these findings the lesion was classified as combined segmental and fundal type. The postoperative course was uneventful, and the patient has been doing well over a one-year follow-up period.

## 3. Discussion

Our literature search revealed only 10 pediatric cases of AMG [2–11]. The clinical features of the eleven pediatric cases (including ours) are presented in Table 1. The median age at the diagnosis was 8 years (5–14 years) in the symptomatic cases. The asymptomatic cases were diagnosed at 12 hours of life and four months of age. The symptoms included abdominal pain, nausea, vomiting, and/or fever. The asymptomatic cases were incidentally diagnosed by US during surveillance for other congenital diseases. Laboratory data were normal in them with the exception of two cases with a past history of slight elevation of serum gamma-glutamyl transpeptidase or liver enzymes. The imaging studies in the reported cases included US (*n* = 10), magnetic resonance imaging (MRI) and/or MRCP (*n* = 6), and CT (*n* = 2). Symptomatic cases were treated by cholecystectomy in with favorable outcomes. Close observation was continued for the asymptomatic cases, and resolution of the lesion was noted eight months later in Case 5, with the same findings persisting for three months in Case 6.

AMG has generally been morphologically classified into three types in adults: segmental, fundal (focal or localized), and diffused (generalized) [1, 12]. Segmental type is the most common type, which is located in the body of the gallbladder, and separates the gallbladder into two communicating compartments. Fundal type is limited to the fundus of the gallbladder with a central dimple located at the tip. Diffused type is the thickening of the entire gallbladder wall. Ootani et al. reported that there were cases of the segmental type combined with fundal type [13]. Interestingly, the thickened gallbladder wall of our case extended over more than distal half of the body, with slight stenosis in the middle of the body, which would be classified as the combined fundal and segmental type. Thus, CT in our case visualized a confusing tumor-like lesion. In contrast, US was quite useful for making a correct diagnosis. The US findings of AMG generally included gallbladder wall thickening and intramural diverticula containing small cystic spaces (RASs), including anechoic or echogenic luminal content in the gallbladder wall. Reverberation artifacts of cholesterol crystals within RASs often show the comet-tail sign [1, 14]. MRI was also useful in our case as it visualized the lining of RASs in the thickened lesion wall as a pearl necklace or rosary sign [1, 14]. When we looked back the CT (Figure 1), the small dot and linear enhancement in the hypodense thick-walled gallbladder could be interpreted as the rosary sign.

The pathogenesis of AMG has been suggested to be as follows: abnormal neurogenic muscular contraction may induce glandular proliferation and hyperplasia of the smooth muscle, leading to RAS formation. There are rare reports of AMG accompanied by PBM in adults [1, 15] and one reported pediatric case (Case 9); however, the causal relationship is unclear. MRCP is necessary to confirm the typical gallbladder abnormality and investigate possible other potential pancreatobiliary disease in children with suspected AMG, such as stone chronic biliary inflammation or pancreatitis.

The reported pediatric cases of AMG included a neonate and a 4-month-old infant (Cases 5 and 6). This indicates that pediatric AMG might in part be developing congenitally. However, the former patient's lesion spontaneously resolved eight months later, and the US findings of the reported two cases showed several comet-tail signs on the thin wall in the literature, without gallbladder wall thickening (a typical feature of AMG). Thus, the lesions of very young asymptomatic children may have a different etiology from those in older children and adults.

Surgery is mandatory when gallbladder lesions with the abovementioned radiological findings are encountered. Cholecystectomy is a treatment of choice for symptomatic AMG patients, and preoperative imaging studies, including US, CT, and MRI, are necessary for the precise determination of the anatomy. Surgeons should also be involved in the close follow-up of asymptomatic patients.

In conclusion, we presented the first case of a child with combined fundal and segmental-type AMG. Because of the rare appearance of the lesion, it resembled a tumor with CT, but repeated US scans were useful as they led to a correct diagnosis.

## Figures and Tables

**Table 1:** The clinical features of eleven pediatric cases with adenomyomatosis of the gallbladder reported in the literature including our case.

Case	Year	First author	Age	Gender	Type	Chief complaint	Laboratory data	Imaging	Treatment (surgery)	Remarks
1	1998	Alberti	5 yr	M	Localized	Abdominal pain	Normal	US, technetium 99 m HIDA, PTC	Laparoscopic cholecystectomy	Hepatobiliary enzymes elevated in the past

2	2003	Cetinkursun	6 yr	M	Diffuse	Abdominal pain, fever, nausea	Normal	US, CT, MRCP	Open cholecystectomy	

3	2005	Zani	5 yr	M	Segmental	Abdominal pain	Normal	US, MRI	Open cholecystectomy	

4	2008	Akcam	9 yr	F	Diffuse	Abdominal pain	Normal	US, MRCP	Open cholecystectomy	

5	2014	Alpati	Neonate	F	No data	—	—	US	—	Congenital heart disease

6	2014	Zarate	4 months	F	Localized	—	—	US	—	Beckwith–Wiedemann syndrome

7	2016	Parolini	11 yr	M	Diffuse	Abdominal pain, nausea, vomiting	GGT elevation	US, MRI	Laparoscopic cholecystectomy	

8	2016	Eroglu	8 yr	F	Diffuse	Abdominal pain, nausea, vomiting	Normal	US	Open cholecystectomy	

9	2016	Eda	8 yr	F	Localized	Abdominal pain	Normal	MRCP, ERCP	Open cholecystectomy	Pancreaticobiliary maljunction

10	2018	Agrusti	14 yr	F	Segmental	Abdominal pain	Normal	US	Laparoscopic cholecystectomy	

11	2019	Our case	12 yr	M	Fundal and segmental	Abdominal pain	Normal	CT, US, MRI/MRCP	Laparoscopic cholecystectomy	

## Abbreviations

GGT: Gamma-glutamyl transpeptidase
US: Ultrasonography
HIDA: Hepatobiliary iminodiacetic acid
PTC: Percutaneous transhepatic cholangiography
CT: Computed tomography
MRCP: Magnetic resonance cholangiopancreatography
MRI: Magnetic resonance imaging
ERCP: Endoscopic retrograde cholangiopancreatography.
